# Result of cementless total hip arthroplasty in a patient with osteopoikilosis, hip dysplasia and advanced osteoarthritis: a case report

**DOI:** 10.1186/s12891-021-04258-w

**Published:** 2021-04-22

**Authors:** Yao-Yuan Chang, Wei-Hsin Lin

**Affiliations:** 1 Departments of Orthopedic Surgery, National Taiwan University Hospital, No.7, Chung Shan S. Rd, Taipei City, 10002 Taiwan; 2 Departments of Orthopedic Surgery, National Taiwan University Hospital Hsinchu Branch, NO.25, Lane 442, Sec.1, Jingguo Rd, Hsinchu City, 30059 Taiwan

**Keywords:** Osteopoikilosis, Sclerosing bone dysplasia, Developmental dysplasia of hip, And total hip arthroplasty

## Abstract

**Background:**

Osteopoikilosis (OPK) is a rare benign sclerosing bone dysplasia and is often incidentally found on plain radiography. OPK generally does not require treatment. Nevertheless, osteonecrosis or degenerative joint disease can occur in the setting of OPK, and little is known with regard to the longevity of arthroplasty prostheses implanted into OPK-bearing bones.

**Case presentation:**

A 55-year-old male presented with progressive right hip pain in 2012. He was diagnosed with coexisting osteopoikilosis and developmental dysplasia of the right hip with advanced osteoarthritis after a series of imaging studies including radiographs, magnetic resonance imaging (MRI), and bone scan. A cementless total hip arthroplasty was performed to treat his right hip pain. Radiographs at eight-year follow-up showed the prosthetic components were well-fixed. Harris hip score of the patient’s right hip was 93. The patient can walk without assistance and work as a construction worker.

**Conclusion:**

Cementless arthroplasty can be considered in patients with hip arthropathies and co-existing osteopoikilosis. Continued follow-up is required to establish the long-term results.

## Background

Osteopoikilosis (OPK) is a rare and benign sclerosing bone dysplasia [1]. It has an autosomal dominant inheritance pattern, affects 2 per 100,000 individuals worldwide, and can be recognized at any age [1, 2]. OPK is usually asymptomatic and found incidentally by plain radiography. The characteristic radiographic feature is multiple, symmetrically-distributed, round or oval sclerotic bone lesions ranging 2–3 mm in diameter in the skeleton, particularly in the periarticular area of the feet, hands, pelvis, and metaphysis of long bones [3–5]. Radiographically OPK and sclerotic bony metastases can be indistinguishable because both conditions appear as multiple radiopaque lesions in the skeleton. Bone scintigraphy is useful in differentiating OPK from osteoblastic bone metastases because OPK lesions typically do not show increased tracer uptake [6].

Although OPK generally does not require treatment [4], it has been reported 15–20% patients with OPK may experience mild arthralgia and joint effusion [7]. Also other hip pathologies, such as symptomatic degenerative joint disease or osteonecrosis, can develop in patients with OPK. So far little is known regarding the longevity of arthroplasty prostheses implanted into bones bearing OPK lesions.

## Case presentation

**Fig. 1:**
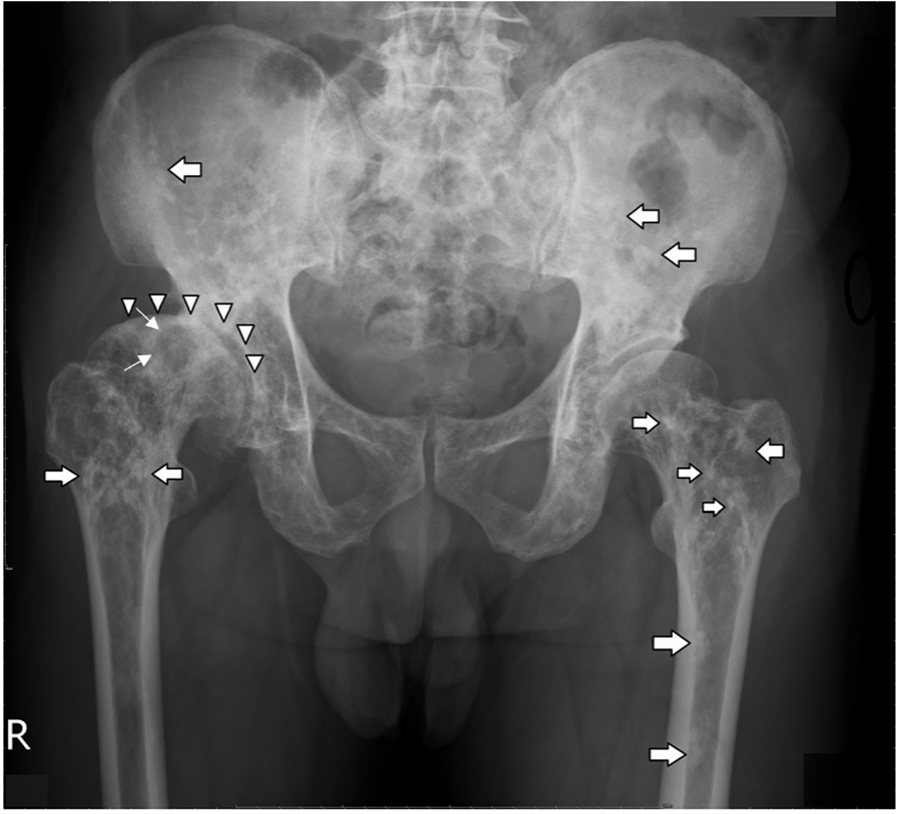
Plain radiography of pelvis. Multiple round and irregular radio-dense bone lesions in the pelvis and bilateral proximal femoral head (arrows). The right hip was typical of adult DDH, with shallow acetabulum and flattened femoral head (arrowheads). The presence of a subchondral cyst was also evident in the right femoral head. (angled arrow)

**Fig. 2:**
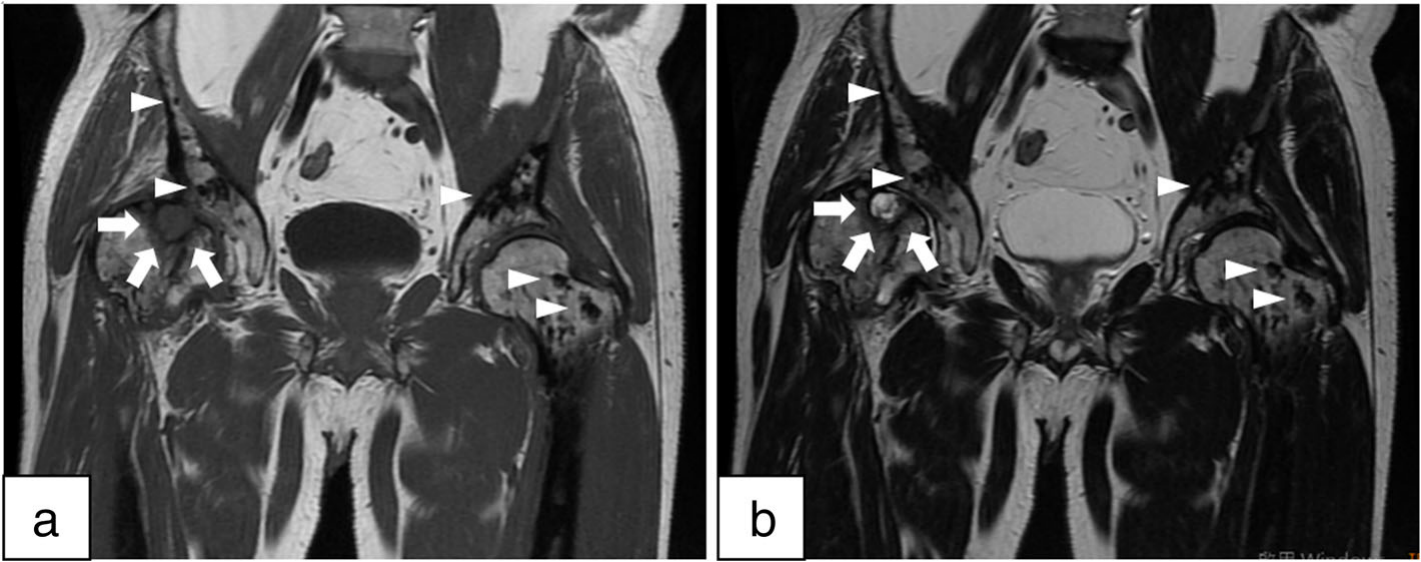
Coronal MRI of Both Hips. A subchondral cyst within the right femoral head, which was low intensity on T1-weighted (**a**) and high intensity with double line sign on T2-weighted images (**b**). (angled arrows). Multiple irregular lesions with low intensity in the pelvis and bilateral proximal femora on T1-weighted and T2-weighted images (**a** and **b**). (arrowheads)

**Fig. 3:**
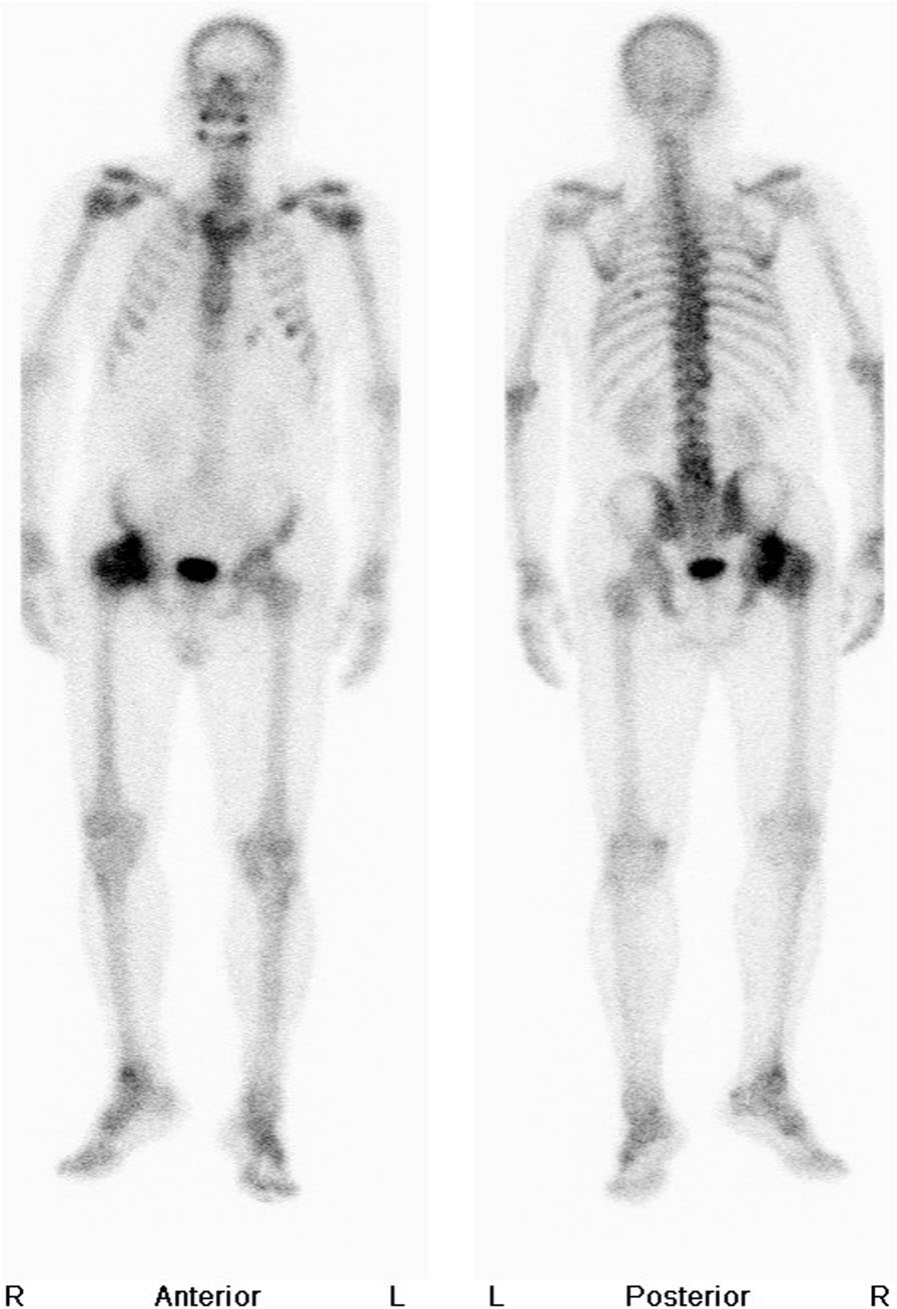
Whole body bone scintigraphy. Abnormal tracer uptake was observed in the right hip joint and right ankle, presumably due to osteoarthritic changes that resulted from years of heavy labor. The left 6th rib also showed increased tracer uptake, which we believe was caused by an old blunt injury to the patient’s left chest

In 2012, a 55-year-old male with diabetes and a hemoglobin A1c level ranging from 6.5 to 6.8% in the past year presented to our orthopedic clinic with progressive right hip pain for 1 month. Detailed history-taking revealed that he had been bothered by vague pain in the right hip since 4 months ago, and had undergone weekly chiropractic manipulation in the past 3 months. The pain worsened significantly 1 month ago, and now was limiting his level of activity and distance of walking. His right hip had a positive Patrick test and decreased range of motion. Plain radiography of the pelvis (Fig. 1) showed multiple round and irregular radio-dense bone lesions in the pelvis and bilateral proximal femurs (arrows), as well as developmental dysplasia of the right hip (DDH). The lateral center-edge angle of the right hip was 9 degrees, and the acetabulum index was 32 degrees. The right femoral head was mushroom-shaped (arrowheads) and had advanced degenerative changes, e.g., a subchondral cyst (angled arrows), in the right hip joint. Pelvis MRI and whole-body bone scintigraphy were ordered to help differentiate between osteoblastic metastases and sclerosing bone dysplasia. The MRI revealed multiple irregular lesions (arrowheads) with low signal intensity in the pelvic bone and bilateral proximal femur on T1-weighted and T2-weighted images (Fig. 2a, b). There was also a subchondral cyst within the right femoral head (arrows. Figure 2 a, b), which showed low signal intensity on T1-weighted images and high signal intensity with double-line sign on T2-weighted images. On bone scintigraphy, significantly increased tracer uptake was observed in the right hip joint (Fig. 3), but not in the multiple skeletal lesions seen on radiographs and MRI. These findings confirmed the diagnosis of adult DDH with advanced osteoarthritis against a background of co-existing osteopoikilosis.

**Fig. 4:**
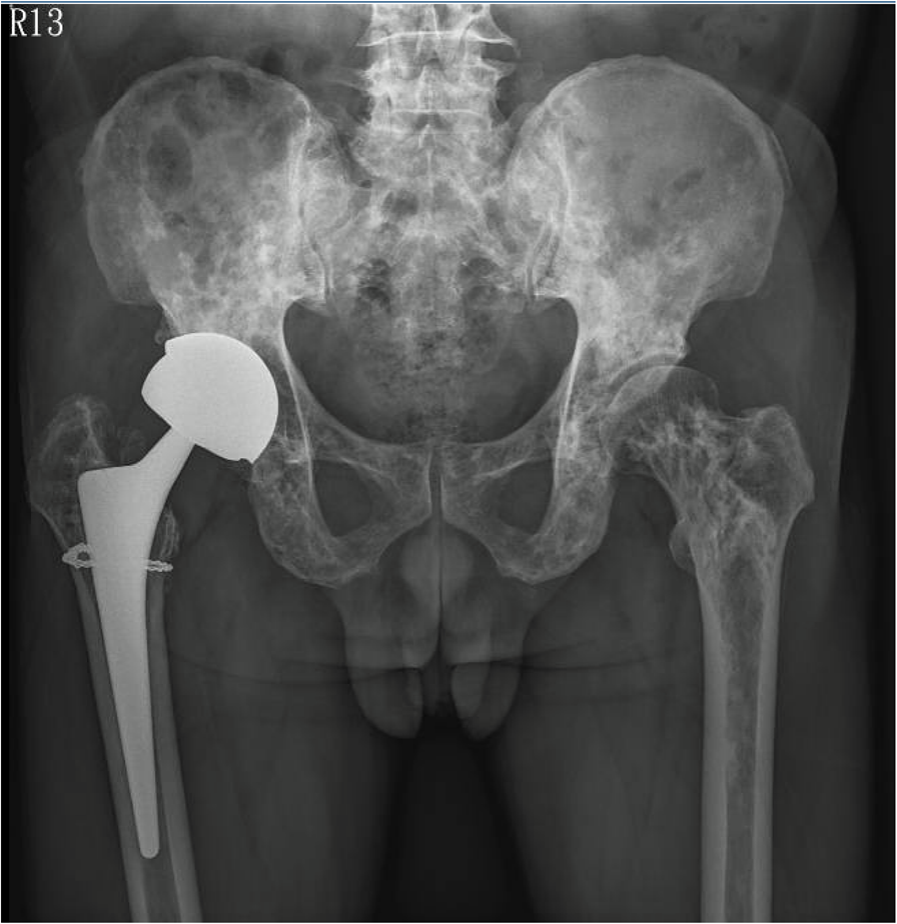
Plain radiography of pelvis. Well-fixed prosthetic components

We hypothesized that osteopoikilosis, a benign sclerosing skeletal dysplasia, would not significantly impair the bone’s biomechanical properties. A cementless large-head metal-on-metal arthroplasty was selected for its supposedly lower dislocation and wear rates. We utilized the standard posterolateral approach with a T-shaped capsulotomy. On reaming of the acetabulum and broaching of the femur, we found the quality of OPK-bearing bone was similar to what one would expect in a healthy middle-aged man. The acetabular component was securely press-fitted using the transverse acetabular ligament as the reference for orientation. We then attempted to put in a proximally fitting femoral stem (Zimmer CLS sportorno stem, Zimmer Inc., Warsaw, IN, USA). The patient’s right femoral neck was excessively anteverted, as is often the case with DDH. Since we did not have any modular hip system (such as the Zimmer Kinectiv or the Depuy S-ROM hip system) in our country at the time of surgery, we aimed to place the femoral stem in a slightly less anteverted position relative to the patient’s native neck anatomy so that it could better match the version of the acetabular cup. Although a properly-sized broach was advanced into the femoral canal, a cortical crack developed on the residual neck during insertion of the femoral prosthesis. Despite this complication, a secure press-fit was still achieved with the femoral stem after a double-stranded wire was applied to stabilize the femur and prevent propagation of the crack. Post-operatively the patient was restricted to toe-touch-weight-bearing ambulation on the right leg for 6 weeks, and placed on a rehabilitation program that included ankle pumping, heel sliding, quadriceps setting, straight leg raising, hip abduction and extension, and glute bridging exercises. After 6 weeks, he began to bear weight to tolerance and ambulate without assistive device. He could walk independently without assistance 3 months after surgery, and returned to work as a construction worker at 4 months post-operatively. Follow-up in the clinic took place every 3 months in the first year, every 6 months in the second year, and annually afterwards. Radiographs at eight-year follow-up showed the prosthetic components were well-fixed (Fig. 4). Harris hip score of the patient’s right hip was 93. The patient continued being asymptomatic for his osteopoikilosis, and still worked as a construction worker.

## Discussion and conclusion

OPK, sometimes called spotted bone disease, is a benign sclerosing bone dysplasia. The etiology and pathogenesis of OPK remain undefined. There are several hypotheses, including: (1) loss-of-function mutations in the LEMD3 gene, causing a generalized fibroproliferative or stenosing disease; (2) hereditary failure to form normal trabeculae along lines of stress; (3) dysplasia of endochondral ossification, affecting resorption or remodeling of secondary spongiosa; and (4) altered osteogenesis [2]. In addition, OPK has been reported as having an autosomal dominant pattern of genetic transmission [8].

OPK and DDH are both a form of skeletal dysplasia, a wide-ranging term used to describe pathologic conditions characterized by disordered development, remodeling and reabsorption of bone and cartilage. Patients with skeletal dysplasia may be predisposed to development of severe degenerative joint disease and need joint replacement at a relatively young age [9]. In our patient, OPK was incidentally found after radiographs were ordered to evaluate symptoms related to DDH and hip osteoarthritis. However, little is known about the longevity and functional outcomes of hip arthroplasty in patients with OPK because of the disease’s rarity. Our search of the literature identified only one article in French by Zahar, et al. describing their successful experience of treating femoral neck fracture in a patient with OPK [10]. The sclerotic bone lesions of OPK consist of dense lamellar osseous tissue that fail to be resorbed [1]. Therefore we theorized that such benign, non-active sclerosing bone lesions would not significantly alter the biomechanical strength of the involved bones, and planned for a cementless total hip arthroplasty. Indeed, in this particular patient, we found that OPK lesions did not cause difficulty with standard reaming of the acetabulum and broaching of the femoral canal. Also the acetabular and femoral components could both be press-fitted in the usual fashion. It is worth noting the extent and severity of sclerotic lesions at the site of prostheses placement could have a bearing on the surgical plan. Although we did not obtain a CT scan of the pelvis and femur at the time, we believe that CT would help determine the most affected area(s) and/or the percentage of OPK lesions in the surgical site. If OPK lesions are abundant or located in areas that might hamper prosthesis placement, the surgeon should be alerted to the possibility of using power tools, such as a high-speed burr, to properly prepare the involved bone(s).

Making a correct diagnosis of OPK is important. The characteristic radiographic feature of OPK is multiple round or oval bone lesions scattered through axial and appendicular skeleton, a picture that resembles osteoblastic metastases, mastocytosis, or tuberous sclerosis [3–5]. Since OPK lesions are composed of mature dense bone, they appear small and dark on both T1 and T2 weighted images on MRI [11]. Bone scintigraphy is useful in distinguishing OPK form osteoblastic metastases because OPK lesions usually do not have enhanced tracer uptake due to inactive bone remodeling.

In conclusion, OPK is a benign sclerosing bone dysplasia that may or may not coexist with other joint abnormalities. Bone scintigraphy plays a critical role in differentiating OPK from osteoblastic metastasis. Our experience suggested that cementless total hip arthroplasty in patients with OPK could achieve satisfactory mid-term functional and radiographic outcomes. Continued follow-up is needed to determine the long-term results.

## Data Availability

The datasets used and/or analyzed during the current study are available from the corresponding author on reasonable request.

## Abbreviations

MRI: Magnetic resonance imaging
OPK: Osteopoikilosis
THA: Total hip arthroplasty
